# The Effect of Retinoic Acid on *in vitro* Maturation and Fertilization Rate of Mouse Germinal Vesicle Stage Oocytes

**Published:** 2011-04-21

**Authors:** Ebrahim Nasiri, Reza Mahmoudi, Mohammad Hadi Bahadori, Iraj Amiri

**Affiliations:** 1. Anatomy Department, Faculty of Medicine, Guilan University of Medical Sciences, Rasht, Iran; 2. Cellular and Molecular Research Center, Faculty of Medicine, Guilan University of Medical Sciences, Rasht, Iran; 3. Cellular and Molecular Research Center, Yasouj University of Medical Sciences, Yasouj, Iran; 4. Research Center for Molecular Medicine, Hamedan University of Medical Sciences, Hamedan, Iran; 5. Anatomy Department, Faculty of Medicine, Hamedan University of Medical Sciences, Hamedan, Iran

**Keywords:** Mouse Oocyte Maturation, In vitro Fertilization, Retinoic Acid

## Abstract

**Objective:**

Retinoids are recognized as important regulators of cell differention and tissue function. Previous studies, performed both in vivo and *in vitro*, indicate that retinoids influence several reproductive events. In this study, we investigated the effect of all-trans retinoic acid (t-RA) on maturation and fertilization rate of immature oocytes (germinal vesicle).

**Materials and Methods:**

Germinal vesicle (GV) oocytes were recovered from 4-6 week old female mice 48 hours after injection of 10 IU pregnant mare serum gonadotropin (PMSG). Collected oocytes were divided into seven groups: control, sham and five experimental groups. t-RA at concentrations of 1, 2, 4, 6, 8 µM were added to oocyte maturation medium in the experimental groups. The maturation rate was recorded after 24 hours of culture in a humidified atmosphere of 5% CO_2_ at 37℃. Fertilization and developmental rates of matured oocytes were recorded after *in vitro* fertilization (IVF) and 24 hour culture.

**Results:**

The rate of oocytes that developed to the metaphase ІІ stage of maturation significantly increased with 2 and 4 µM t-RA compared to the control and sham groups (p<0.05). In addition, the number of fertilized oocytes was significantly higher in 4 µµ retinoic acid compared to the control (p<0.05), but the difference between the number of fertilized oocytes which developed to the 2-cell stage was not significant between the two groups.

**Conclusion:**

The results show that t-RA enhanced mouse oocyte maturation *in vitro* and improved fertilization and development rates in a dose dependent manner.

## Introduction

*in vitro* maturation of oocytes offers an alternative techinqe to obtain mature oocytes in cases unresponsive to hormonal stimulation or those at risk of ovarian hyperstimulation (1-3). However, *in vitro* maturation of human immature oocytes exhibit acceptable meiotic competence to metaphase ΙΙ (MΙΙ), but their subsequent developmental competence remains disappointingly low. Only 40%-80% of fertilized *in vitro* matured oocytes progress through early cleavage, and of those that do cleave and are transferred, 15% implant to form a viable fetus (4-6). Oocyte maturation is often conceptually divided into nuclear and cytoplasmic processes. Nuclear maturation is a term that refers to the resumption of meiosis from the germinal vesicle (GV) stage and progression to MΙΙ. Cytoplasmic maturation is a more general term that refers to other maturational events (not directly related to meiotic progression) that prepare the oocyte for fertilization and preimplantation development (5). Despite many reports about successful maturation and development of mammalian immature oocytes *in vitro* (7-10), the quality of maturation appears to be suboptimal because embryos resulting from *in vitro* matured oocytes show more frequent cleavage blocks and overall retarded cleavage rates compared to oocytes matured in vivo (11, 12). It is known that insufficient cytoplasmic maturation of the oocyte fails to promote male pronuclear formation and will thus increase chromosomal abnormalities after fertilization (13). Therefore, developing an optimal culture system is essential to improve the quality of oocytes matured *in vitro*. An important method to improve oocyte quality is the supplementation of maturation media by growth factors, cytokines and vitamins.

There is some evidence about the roles of vitamin A (retinol) and its active derivatives (i.e., retinoids) in very early events of mammalian reproduction, including follicular growth and oocyte maturation, and embryonic growth and development. For example, the concentration of retinol in bovine follicular fluid has been shown to be an indicator of follicular quality and was highest in healthy follicles, lowest in atretic follicles and highly correlated with estradiol concentrations (14-16). Retinol or β-carotene administration has been shown to prevent fetal resorption in rats (17), increase the number of births in rabbits, sow, mice and bovines (18-20), and increase litter size in swine (9). Retinol administration to ewes, in combination with superovulation has been shown to improve the competence of resultant 1-4 cell and morula stage embryos collected from the oviduct and uterus, respectively, to develop to the blastocyst stage when cultured *in vitro* (19). In cattle, retinol injections improved the estimated quality of embryos collected from superovulated animals but did not increase the number recovered (21). Also, one study demonstrated that retinoic acid exerts an adverse effect on mouse embryo growth during early post-implantation development (21).

Studies performed *in vitro* on the effects of vitamin A metabolites used in some assisted reproductive techniques (ART), including superovulation, ovum pick up and *in vitro* maturation, have provided evidence for the specific roles of vitamin A in oocyte cytoplasmic maturation (20). According to these findings, it seems that the use of vitamin A in culture media during *in vitro* maturation of mamalian oocytes can enhance the rate of oocyte maturation and their quality.

Although, the positive effects of vitamin A on cytoplasmic maturation of bovine oocytes has been shown previously (20-23), insufficient data exists about the effects of vitamin A and its derivites on oocyte maturation in other species.

According to the mentioned data, this study investigated the effects of all-trans retinoic acid (t-RA) on *in vitro* maturation, fertilization and developmental rates of mouse immature oocytes *in vitro*.

## Materials and Methods

All experiments were performed according to the Iranian Council for Use and Care of Animal Guidelines and approved by the Animal Research Ethical Committee of Guilan University of Medical Sciences.

### Reagents and Media

All chemicals were purchased from Sigma Chemical Company, unless otherwise indicated. t-RA was dissolved in 100% ethanol, appropriate dilutions made, and aliquots stored at -80℃ until use.

### Collection of immature mouse oocytes

Oocytes were obtained from 4-6 week NMRI female mice. The animals were kept under controlled conditions (12 hour light:12 hour dark), fed with water and pellets ad libitum. Mice were stimulated by an i.p. injection of 10 IU pregnant mare serum gonadotropin (PMSG). The animals were killed 45 hours later by cervical dislocation and their ovaries placed in TCM-199 culture media supplemented with 10% fetal bovine serum (FBS). Immature oocytes in the germinal vesicle stage (GV stage) were released by puncturing the follicles with a 28 G sterile needle under a stereomicroscope. A total of 1145 oocytes were obtained from 42 ovaries and used for *in vitro* maturation. The average number of collected oocytes was 19.8 per ovary.

### In vitro maturation (IVM)

**Fig 1:**
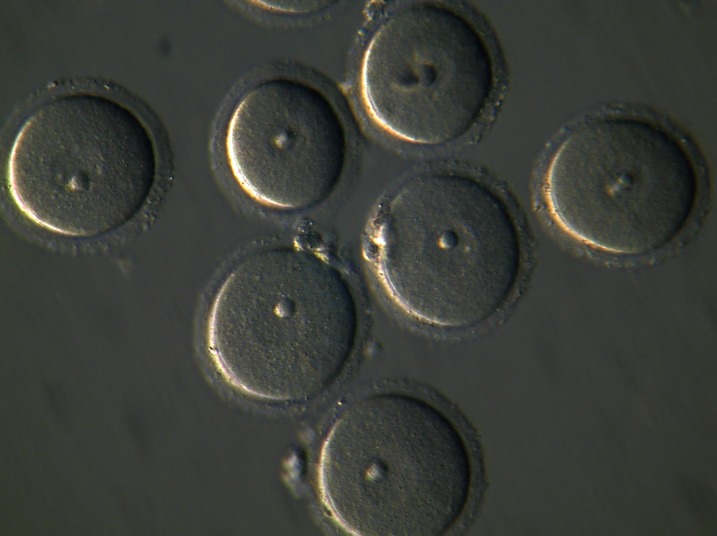
The collected germinal vesicle (GV) oocytes before in vitro maturation (Magnification: ×200).

The collected GV-stage oocytes (Fig 1) were randomly divided into control, sham and five experimental groups. Each group was placed in 25 µl micro drops of maturation medium that consisted of TCM-199 supplemented with 10% FBS, 50 mg/l streptomycin, 60 mg/l penicillin and 1 µg/l epidermal growth factor (EGF), over laid with embryotested light mineral oil and incubated for 24 hours in a humidified atmosphere of 5% CO_2_ at 37℃. In experimental groups, t-RA at concentrations of 1, 2, 4, 6 and 8 µµ dissolved in pure ethanol was added to the maturation medium. In the sham group, ethanol alone 0.2% (v/v) was added to the maturation medium. After 24 hours incubation, oocytes were observed by an inverted microscope. Morphological changes in the nucleus or extrusion of the first polar body (MΙΙ) were used at the criterion for nuclear maturation of GV stage oocytes. Matured oocytes were collected and used for in vitro fertilization.

### In vitro fertilization (IVF)

Sperm were collected from the epididymes of NMRI male mice, aged 12 weeks. The sperm suspension (1×10^6^ motile spermatozoa/ml) was capacitated for 2 hours in 1 ml T6 culture medium that contained 5 mg/ml bovine serum albumin (BSA) fraction V. *in vitro* matured oocytes from each group were placed into 0.9ml droplest of T6 to which 0.1 ml capacitated spermatozoa was added. After 5 hours incubation, the oocytes were washed through three droplets of T6 medium and checked for extrusion of the second polar body and formation of male and female pronuclei, which indicated fertilization. Then, oocytes were cultured in fresh droplets of T6 (25 µl) under mineral oil and assessed for cleavage to the 2-cell stage after 24 hours.

### Statistical analysis

Collected data were analyzed by the chi-square test. The differences in the values of maturation, fertilization and developmental rates were considered significant when p<0.05.

## Results

### In vitro maturation of mouse oocytes

**Fig 2:**
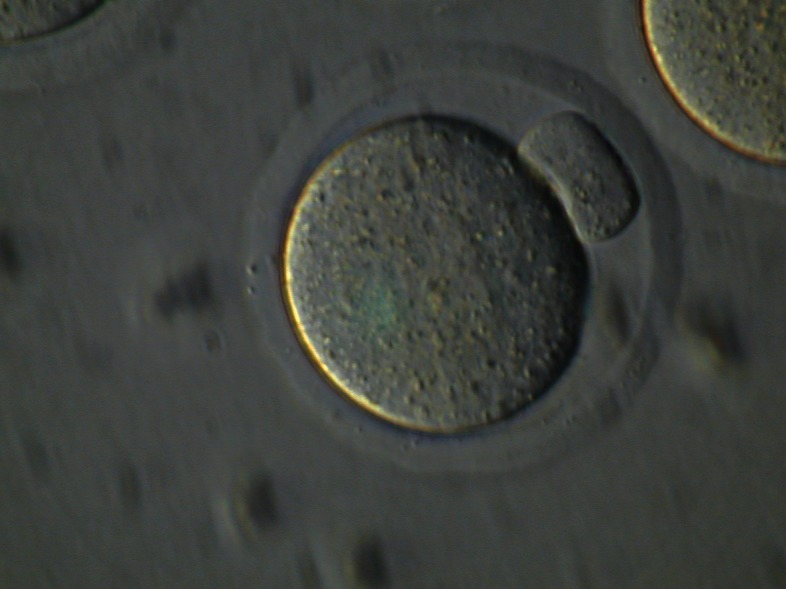
Final stage of oocyte maturation, metaphase II (MII) oocyte after 24 hours culture in vitro (Magnification: ×200).

Table 1 shows the number of oocytes that attained the MΙΙ stage (Fig 2) after 24 hours of culture. The maturation rate of oocytes in groups treated with 2 µµ and 4 µµ retinoic acid (groups 2 and 3) were significantly higher than the control and sham groups (p<0.05). The degeneration rates in groups 4 and 5 were significantly higher than other groups.

### IVF and development of mouse oocytes

**Fig 3:**
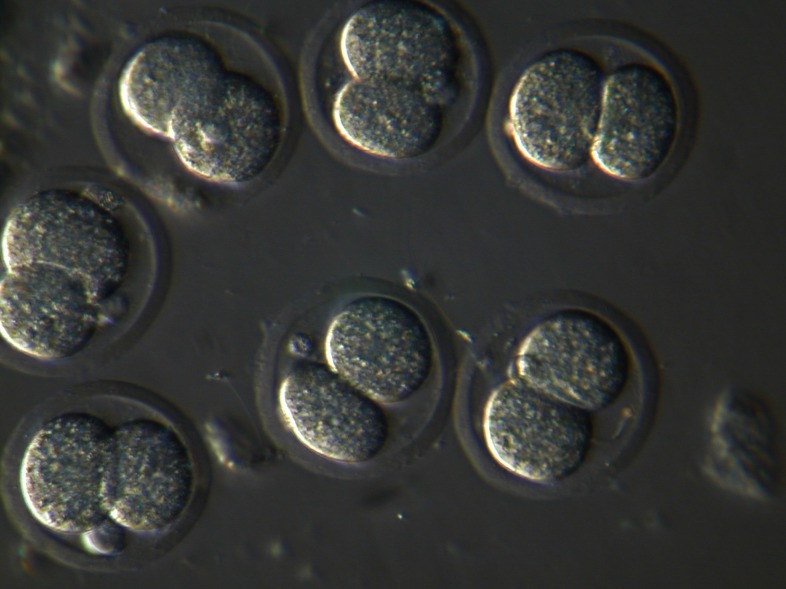
Cleaved (2-cell) embryos at 24 hours post-fertilization (Magnification: ×200).

As shown in table 1, the rate of fertilization in oocytes treated with 4 µµ retinoic acid were significantly higher than those of the control group (p<0.05). However, the difference between the percent of fertilized oocytes which developed to the 2-cell stage (Fig 3) was not significant (p>0.05) between the two groups.

**Table 1:** Maturation rate of mouse oocytes after 24 hours culture


Groups	All-trans retinoic acid dose (µM)	No. of GV stage oocytes	No. of GVBD (%)	No. of MII (%)	No. of undeveloped and degenerated oocytes (%)

Control	0	182	22 (12.08)	115 (63.18)	45 (24.72)
Sham	0 (ethanol)	163	18 (11.04)	99 (60.73)	46 (28.22)
Group 1	1	166	15 (9.03)	114 (68.67)	37 (22.28)
Group 2	2	158	20 (12.65)	112 (70.88)	26 (16.45)
Group 3	4	164	13 (7.92)	119 (72.56)	32 (19.51)
Group 4	6	160	16 (10)	91 (56.87)	53 (33.12)
Group 5	8	152	21 (13.81)	81 (53.28)	50 (32.89)


GV=Germinal vesicle oocyte, GVBD=Germinal vesicle breakdown, M II=Metaphase II

**Table 2:** Fertilization and developmental rates of mouse oocytes in control and experimental groups


Groups	No. of MII	Fertilized oocytes (%)	2-cell stage embryos (%)	Non-fertilized and degenerated oocytes (%)

Control	115	76 (66.08)	45 (39.13)	39 (33.91)
Sham	99	62 (62.62)	34 (34.34)	37 (37.37)
Group 1	114	74 (64.91)	48 (42.1)	40 (35.08)
Group 2	112	88 (78.57)	58 (51.78)	24 (21.42)
Group 3	119	86 (72.26)	57 (47.89)	33 (27.73)
Group 4	91	52 (57.14)	30 (37.97)	39 (42.85)
Group 5	81	43 (53.08)	26 (32.09)	38 (46.91)

## Discussion

In the present study, we used mouse immature oocytes to evaluate the effects of retinoic acid on maturation, fertilization and embryonic development to the 2-cell stage *in vitro*. Retinoic acid administration during the maturation period alone resulted in concentration-dependent effects. Whereas the presence of 1 µµ retinoic acid had no effect on *in vitro* maturation and development, 2 µµ and 4 µµ retinoic acid tended to improve maturation and rates of development compared to the other groups. At a concentration of 6 µµ and 8 µµ, retinoic acid significantly reduced maturation, fertilization and developmental rates compared to the other groups. These results indicated the effect of vitamin A on oocyte maturation. Previously, it has been shown that vitamin A has an essential role in the physiology of vertebrates, being involved in cell growth and differentiation, embryonic development and vision. The retinoids are a large family of natural and synthetic compounds related to vitamin A (t-RA). High vitamin A concentrations may be teratogenic to the embryo. However it has been confirmed that both vitamin A deficiency and high concentrations of retinoid are associated with developmental abnormalities by altering the normal relationship between cellular retinoid levels and the embryonic genetic developmental program (24). The vitamin A derivative, retinoic acid, is the most relevant retinoid during vertebrate development and acts on cells to establish or change the pattern of gene activity. This retinoid could influence cytoplasmic maturation and the subsequent capacity of the oocyte to progress developmentally (25).

Whereas it has been suggested that the requirement for vitamin A activity in the embryo begins at the time of organization, there is evidence that the oocyte’s developmental competence could be enhanced by retinoid support during oocyte intrafollicular growth, oocyte maturation and embryonic development (23-25).

The beneficial effect of vitamin A during oocyte growth in vivo can be reproduced by retinol derivatives and added to an *in vitro* culture system into which the oocytes are meiotically arrested (22).

The obtained data in this study are comparable with previous reports about the effects of vitamin A on mammals. For example, it has been reported that retinol administration to donor animals improved embryonic quality in both superovulated sheep (9), cows (10) and in non-superovulated gilts (26). Also, addition of retinol metabolite 9-cis retinoic acid to maturation culture media could promote cytoplasmic maturation of oocytes and subsequent early embryonic development in bovines (20, 22, 25) and mice (26).

However, the possible mechanisms of the positive effects of retinoic acid on oocytes are hypotheses, but retinoic acid may promote cytoplasmic maturation of oocytes via its modulatory effects on the gene expression of gonadotropin receptors, midkine, cyclooxygenease-2 and nitric oxide syntheses in cumolose-granolosa cells (1). As maturation proceeds, the cytoplasmic granules migrate to the cortex and occupy the area just beneath the oolemma, at same time the nucleus enters the MII stage. Cortical granule migration is a common phenomenon in mammalian oocytes during maturation both in vivo and *in vitro*. This migration is associated with a gain in developmental competence by the oocyte and blocks polyspermy once migrated cortical granule contents are released. The most relevant finding within our cortical granules study was probably that RA induced cortical granules prior to maturation. Also, the cortical granules distribution after retinoic acid exposure formed a uniform monolayer beneath the oolemma with lesser clustering once RAprematured oocytes were allowed to mature in the absence of retinoic acid (22). Taken together, these results suggest a role for retinoic acid in the improvement of developmental competence of oocytes. However, the exact timing (and possibly also the concentration) of retinoic acid exposure is critical since it alters the normal retinoic acid migration and distribution.

## Conclusion

This study suggests that retinoic acid increases oocyte maturation, fertilization and embryo developmental rates in mice. Despite beneficial effects of retinoic acid on oocyte maturation and fertilization, it is strongly recommended that more animal studies should be undertaken to evaluate its safety, with particular attention to its potential teratogenic effects and the long-term outcome of offspring, before it is applied to human-assisted reproductive programs.
